# Neutrophil Lymphocyte Ratio and Cardiovascular Disease Risk: A Systematic Review and Meta-Analysis

**DOI:** 10.1155/2018/2703518

**Published:** 2018-11-11

**Authors:** Teeranan Angkananard, Thunyarat Anothaisintawee, Mark McEvoy, John Attia, Ammarin Thakkinstian

**Affiliations:** ^1^Section for Clinical Epidemiology and Biostatistics, Faculty of Medicine, Ramathibodi Hospital, Mahidol University, Bangkok, Thailand; ^2^Division of Cardiovascular Medicine, Department of Medicine, Faculty of Medicine, HRH Princess Maha Chakri Sirindhorn Medical Center, Srinakharinwirot University, Nakhon Nayok, Thailand; ^3^Department of Family Medicine, Faculty of Medicine, Ramathibodi Hospital, Mahidol University, Bangkok, Thailand; ^4^Center for Clinical Epidemiology and Biostatistics, The School of Medicine and Public Health, The University of Newcastle, Newcastle, NSW, Australia

## Abstract

**Objective:**

This systematic review aimed to measure the association between neutrophil lymphocyte ratio (NLR) and cardiovascular disease (CVD) risk.

**Methods:**

Relevant studies were identified from Medline and Scopus databases. Observational studies with NLR as a study factor were eligible for review. The outcomes of interest were any type of CVD including acute coronary syndrome, coronary artery disease, stroke, or a composite of these cardiovascular events. Mean differences in NLR between CVD and non-CVD patients were pooled using unstandardized mean difference (USMD). Odds ratios of CVD between high and low NLR groups were pooled using a random effects model.

**Results:**

Thirty-eight studies (n=76,002) were included. High NLR was significantly associated with the risks of CAD, ACS, stroke, and composite cardiovascular events with pooled ORs of 1.62 (95% CI: 1.38-1.91), 1.64 (95% CI: 1.30, 2.05), 2.36 (95% CI: 1.44, 2.89), and 3.86 (95% CI: 1.73, 8.64), respectively. In addition, mean NLRs in CAD, ACS, and stroke patients were significantly higher than in control groups.

**Conclusion:**

High NLR was associated with CAD, ACS, stroke, and composite cardiovascular events. Therefore, NLR may be a useful CVD biomarker.

## 1. Introduction

Cardiovascular diseases (CVD) are the leading causes of mortality worldwide [1, 2]. Approximately 17.7 million people died from CVD in 2015, with one-third due to coronary heart disease (CHD) and stroke. In addition, CVD carries a high economic burden, costing about $316.1 billion/year [3]. Therefore, risk stratification and prognostication in CVD are important so that individuals at high risks can be accurately targeted for prevention.

The inflammatory response is a key mechanism in the pathogenesis of atherosclerosis and its progression [4]. Neutrophils secrete inflammatory mediators that can cause vascular wall degeneration. Conversely, lymphocytes regulate the inflammatory response and thus have an antiatherosclerotic role. Therefore, the neutrophil to lymphocyte ratio (NLR) has been proposed as an inflammatory biomarker [5] and potential predictor of risk and prognosis in CVD.

A previous systematic review suggested prognostic impacts of NLR on all-cause mortality and recurrent cardiovascular events (CVEs) among CHD patients [6–8]. However, the effect of NLR on CVD is still unclear and thus far, there has been no meta-analysis quantifying these associations. We therefore conducted a systematic review and meta-analysis of observational studies aiming to explore and quantify the association between NLR and CVD risk.

## 2. Methods

A systematic review of observational studies was performed according to the MOOSE guidelines [9]. This review has been registered in PROSPERO (no. CRD42016043554).

### 2.1. Search Strategy

Relevant studies were identified from Medline and Scopus databases since their inceptions to 19th August 2018. The following search terms were used: ‘cardiovascular disease', ‘coronary heart disease', ‘coronary artery disease', ‘myocardial infarction', ‘angina', ‘stroke', ‘cerebrovascular', and ‘neutrophil lymphocyte ratio'. The search strategies for both databases are presented in Supplementary Appendix. Reference lists of included studies and previous systematic reviews were also explored to identify eligible studies not located using the database searches.

### 2.2. Selection of Studies

Identified studies were independently selected based on titles and abstracts by two reviewers (T.A.1 and T.A.2). Full articles were retrieved if a decision could not be made from the titles and abstracts. Disagreements were resolved by consensus and discussion with a third party (A.T.). Inclusion criteria were as follows: (1) any type of observational study or baseline randomized controlled trial published in English; (2) including adult patients aged ≥18 years; (3) having NLR as a study factor; (4) having CVD as outcome of interest; (5) providing sufficient data for pooling, i.e., number of patients, mean and standard deviation of NLR between CVD and non-CVD patients, and/or numbers of contingency cells between low/high NLR and CVD/non-CVD. For studies with insufficient data, up to 3 attempts to contact corresponding authors were made.

### 2.3. Outcome Measurement

The outcomes of interest were coronary artery disease including chronic stable angina, acute coronary syndrome, cerebrovascular diseases including ischemic/hemorrhagic stroke or transient ischemic attack, and cardiovascular (CV) death, as well as a composite CVD outcome of these.

### 2.4. Study Factor

The study factor was NLR, measured from the complete blood count according to the original studies, and was calculated by dividing neutrophil count by lymphocyte count.

### 2.5. Data Extraction

All data were independently extracted by 2 reviewers (T.A.1 and T.A.2). Discrepancies between two reviewers were resolved through discussion with the senior author (A.T.). Characteristics of the included studies [i.e., study design, setting, mean age, body mass index (BMI), and mean NLR of study participants, percentages having diabetes mellitus, hypertension (HT), dyslipidemia (DLP), and smoking] and cut-off values of NLR for studies assessing the effect of high versus low NLR were extracted. Incidence or prevalence of CVD and non-CVD patients between high and low NLR was extracted for pooling odds ratio (OR). For studies that did not provide these estimates, risk ratios [e.g., OR, relative risk (RR), and HR] along with their 95% confidence intervals (CIs) were extracted instead. To complete data analysis of continuous variables data, the mean difference was calculated and used as one of the summary effect sizes; the number of patients and mean and standard deviation (SD) of NLR between CVD and non-CVD patients were extracted for pooling mean difference of NLR.

### 2.6. Risk of Bias Assessment

Risk of bias assessments of included studies were independently assessed by two reviewers (T.A.1 and T.A.) using the Newcastle–Ottawa quality assessment scale [49]. NOS has three domains to assess, which are (1) selection of study groups (4 items), (2) comparability of groups (2 items), and (3) ascertainment of exposure and outcome (3 items). Each item in the 3 domains was graded as 0 to 1 with a total score ranging from 0 to 9; higher total score reflected higher quality or lower risk of bias.

Since the NOS does not have criteria for judging cross-sectional studies, criteria for cohort study were adapted to assess the risk of bias for cross-sectional study. Two items in the domain of ascertainment of outcome (i.e., adequate duration of follow-up and adequate follow-up of cohort) were excluded because they are not relevant for cross-sectional studies. Therefore, the total score for this design ranged from 0 to 7, instead of 0 to 9.

### 2.7. Statistical Analysis

For comparison of mean NLR between CVD and non-CVD groups, mean differences in NLR between CVD and non-CVD groups were estimated and were pooled using the unstandardized mean difference (USMD). For categorical outcomes, cut-off points of NLR for each study were recategorized into low versus high NLR groups as follows: For those studies with four NLR categories, two lower and two higher NLR categories were classified as low and high NLR groups, respectively; for three categories, the low and intermediate groups were combined and assigned as low NLR whereas the last group was assigned as high NLR. Odds ratio of having CVD between high and low NLR groups of each study was then estimated and pooled using a fixed effects model (inverse variance method) if there was no heterogeneity between studies; otherwise, a random-effects model (DerSimonian and Laird) was applied.

Cochrane's Q test and the degree of heterogeneity (I^2^ statistic) were applied to assess heterogeneity between studies. Heterogeneity between studies was considered, if P-value from Cochrane's Q test was less than 0.10 or degree of heterogeneity was equal to or greater than 25%. Sources of heterogeneity were explored by considering the covariables (i.e., mean age, BMI, and smoking history) one by one in a meta-regression model. Subgroup analysis was further performed according to the covariables which could decrease Tau^2^ greater than 50%.

Publication bias was explored using Egger's test and funnel plot. If there was asymmetry of funnel plot, a contour enhanced funnel plot was applied to explore the cause of asymmetry.

All analyses were performed using STATA software, version 15.0 (StataCorp LP, College Station, TX, USA). A two-sided test with P-value < 0.05 was considered for statistical significance except for the heterogeneity test, in which P-value < 0.1 was applied.

## 3. Results

**Figure 1:**
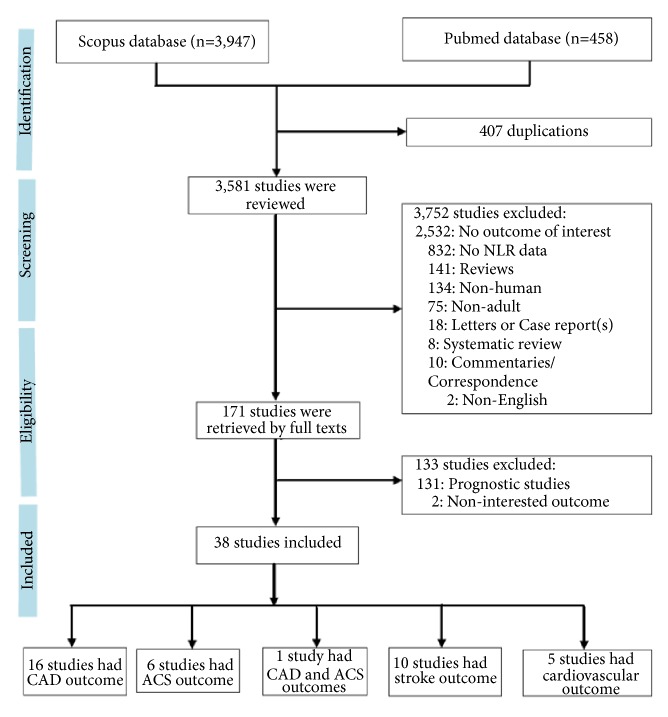
Flow chart of study selection.

A total of 4,405 relevant studies were identified from Medline and Scopus databases; see Figure 1. Among them, 4,367 studies were excluded leaving 38 studies [10–47] eligible for review. These consisted of 76,002 participants from 11 case–control, 9 cohort, and 18 cross-sectional studies. There were 16, 6, 10, and 5 studies that, respectively, reported stable CAD [10–17, 19–26], ACS [27–32], stroke [33–42], and composite CVD outcomes [43–47]. One study [18] reported both CAD and ACS.

Characteristics of included studies are illustrated in Table 1. Mean age of study participants ranged from 34.9 to 73.2 years. Some studies focused on patients with specific diseases including diabetic patients in 6 studies [14, 16, 23, 30, 43, 44], gastric cancer in 1 study [40], CKD patients in 2 studies, and HIV patients in 1 study [47]. Percentages of patients having DM, HT, and DLP and smoking ranged within 0%-100%, 5.9%-80.9%, 12.4%-83.9%, and 0%-45.8%, respectively.

### 3.1. Risk of Bias Assessment

Results of the risk of bias assessments are presented in Supplementary Tables 1 and 2. The total scores ranged from 4 to 7, 6 to 8, and 3 to 7 for case-control, cohort, and cross-sectional studies, respectively. For case-control study, nearly all studies had high risk of bias for definition of case and all for nonresponse rate but had low risk of bias for representativeness of cases, definition of controls, assessment of exposure, and same method of outcome ascertainment for cases and controls. For cohort and cross-sectional studies, almost all studies had low risk of bias for ascertainment of exposure and assessment of outcome.

### 3.2. CAD Outcome

Among 17 studies of 8,988 subjects, 4 studies [14, 16, 20, 23] reported ORs of high versus low NLR, 8 studies [10–12, 17, 19, 21, 22, 26] reported mean differences of NLR between CAD and non-CAD patients, and 5 studies [13, 15, 18, 24, 25] reported both.

#### 3.2.1. High versus Low NLR

**Figure 2:**
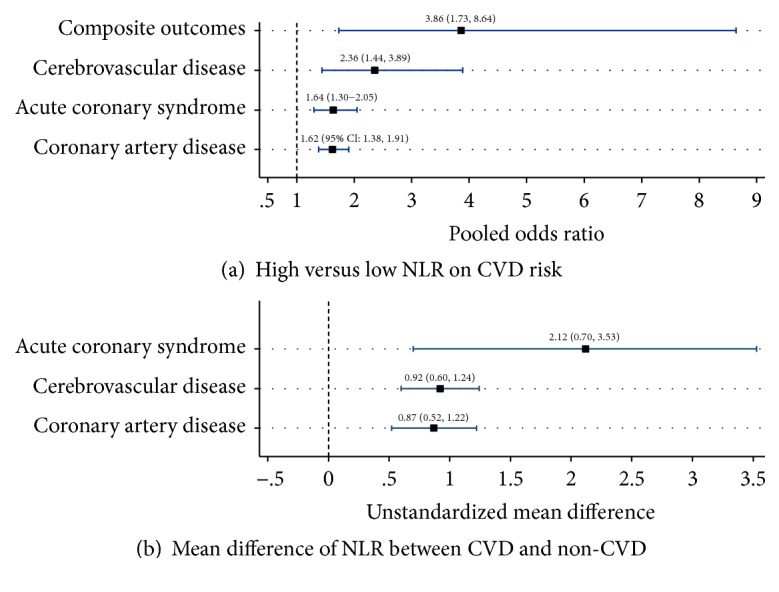
Summary of pooled effect sizes of neutrophil lymphocyte ratio on cardiovascular risk.

A total of 7,405 patients were included in pooling. Study design was cross-sectional in most studies (8/9), while one [18] study was prospective. Contingency data of NLR and CAD groups are presented in Table 2. The NLR cut-off points ranged from 1.80 to 2.60. Estimated ORs from those 9 studies were moderately varied (Chi-square = 17.01, P-value = 0.03, I^2^ = 53.0% with a pooled OR of 1.62 (95% CI: 1.38-1.91)); see Figure 2(a) and Supplementary Figure 1A.

Sources of heterogeneity were also explored. Only race (Caucasian versus Asian) and age (≤ versus > 65 years) variables could reduce the I^2^ from 53% to 47.31% and 39.74%, respectively, in the meta-regression model. A subgroup analysis was performed and showed that pooled ORs were higher in Asians (1.90; 95% CI: 1.26, 2.87) than Caucasians (1.51; 95% CI: 1.27, 1.79) and greater in patients ≤ 65 years (1.73; 95% CI: 1.44, 2.07) than patients > 65 years (1.43; 95%CI: 1.08, 1.89); see Supplementary Figures 1B and 1C.

#### 3.2.2. Mean Difference of NLR

Thirteen studies reported mean difference in NLR between CAD and non-CAD patients (see Table 3). Most study designs (9/13 studies) were cross-sectional; three were case-control [11, 17, 22] and one [18] was cohort. USMD was 0.87 (95% CI: 0.52, 1.22). Heterogeneity test and I^2^ suggested high heterogeneity between studies (Chi-square = 611.32; P-value <0.001; I^2^ = 98.0%); see Figure 2(b) and Supplementary Figure 2A. Possible sources of heterogeneity were explored in a meta-regression, but none of them could decrease the degree of heterogeneity. There was no evidence of publication bias from Egger's test (coefficient = 0.31; P-value = 0.253), but the funnel plot showed asymmetry; see Supplementary Figure 2B. A contour-enhanced funnel plot was therefore constructed, which suggested that asymmetry was more likely due to heterogeneity between studies; see Supplementary Figure 2C.

### 3.3. ACS Outcome

Among 7 studies of ACS outcome, 2 studies [28, 32] reported OR of high versus low NLR, 2 studies [30, 31] reported mean differences between ACS patients and controls, and 3 studies [18, 27, 29] reported both.

#### 3.3.1. High versus Low NLR

There were 1,816 subjects from 5 studies; see Table 2. Study designs were cross-sectional (n=2), case-control (n=1), and cohort (n=2). The cut-off points for defining high NLR ranged from 2.19 to 5.70. The pooled OR was 1.64 (95% CI: 1.30, 2.05) with low heterogeneity (Chi-square = 4.59; P-value =0.332; I2 = 12.8%); see Figure 2(a) and Supplementary Figure 3A. There was no evidence of publication bias from Egger's test (coefficient = 0.49; P-value = 0.943), a funnel plot and contour-enhanced funnel plot; see Supplementary Figures 3B and 3C.

#### 3.3.2. Mean Difference of NLR

Mean differences of NLR between ACS (n=832) and non-ACS (n=541) patients from five studies are presented in Table 3. Study designs were cross-sectional (n=2), case-control (n=2), and cohort (n=1). The USMD of NLR was 2.12 (95% CI: 0.70, 3.53) with high heterogeneity (Chi-square = 114.63; P-value <0.001; I^2^ = 96.5%); see Figure 2(b) and Supplementary Figure 4A.

None of the covariables reduced the I^2^ after exploring sources of heterogeneity. There was no evidence of publication bias from Egger's test (coefficient = 4.19; P-value = 0.314), a funnel plot and contour-enhanced funnel plot; see Supplementary Figures 4B and 4C.

### 3.4. Stroke Outcome

Among 10 studies of 58,867 participants, 2 studies [33, 39] reported OR of high versus low NLR, 6 studies [35, 37, 38, 40–42] reported mean differences between stroke patients and controls, and 2 studies [34, 36] reported both. Types of stroke were ischemic stroke and TIA.

#### 3.4.1. High versus Low NLR

There were 24,769 stroke patients and 32,977 controls from 4 studies. Two studies [33, 34] included patients with atrial fibrillation (AF). Study designs were case-control (n=2), and cohort (n=2). Cut-off points of NLR ranged from 3.00 to 3.17. The pooled OR was 2.36 (95% CI: 1.44, 3.89) with moderate heterogeneity (Chi-square = 10.99, P-value = 0.012, I^2^ = 72.7%); see Figure 2(a) and Supplementary Figure 5A.

By the meta-regression model, the population variable (AF versus non-AF) could reduce the I^2^ from 72.7% to 48.1%. A subgroup analysis was evaluated and showed that pooled ORs were lower in AF (1.75; 95% CI: 1.14, 2.68) than non-AF (3.83; 95% CI: 1.27, 11.49); see Supplementary Figure 5B. The degrees of heterogeneities (I^2^) were reduced to 40.0% and 54.2% in studies with the AF and non-AF, respectively. There was evidence of publication bias suggested from Egger's test (coefficient = 2.34; P-value = 0.045) and funnel plot; however a contour-enhanced funnel plot suggested heterogeneity rather than publication bias; see Supplementary Figure 5C and 5D.

#### 3.4.2. Mean Difference of NLR

The USMD in NLR between stroke patients (n=633) and controls (n=614) from 8 studies was 0.92 (95% CI: 0.60, 1.24) with high heterogeneity (Chi-square = 121.86; P-value <0.001; I^2^ = 94.3%); see Table 3, Figure 2B, and Supplementary Figure 6A. The source of heterogeneity could not be identified from a meta-regression. There was no evidence of publication bias from Egger's test (coefficient = 0.82; P-value = 0.931), but a funnel plot showed asymmetry; see Supplementary Figure 6B. The contour enhanced funnel plot showed that most studies fell in the significant area and only one study fell in the nonsignificant area, so asymmetry might be due to publication bias; see Supplementary Figure 6C.

### 3.5. Composite Outcomes

#### 3.5.1. High versus Low NLR

Five studies [43–47] were pooled for composite CVEs. Only one study [43] was cross-sectional and the remaining four studies were cohort. Different study endpoints were reported including ACS, stroke, and peripheral artery disease (n=3) [45–47], ischemic stroke and CAD (n=1) [43], and AMI and revascularization (n=1) [44]. Cut-off points used for defining NLR ranged from 1.20 to 3.67. The pooled ORs across studies were 3.86 (95% CI: 1.73, 8.64) with high heterogeneity (Chi-square = 29.97; P-value =0.001; I^2^ = 86.7%); see Table 2, Figure 2(a), and Supplementary Figure 7A. Sources of heterogeneity were explored but none could reduce the I^2^. However, sensitivity analysis was performed according to the length of follow-up. Follow-up time ranged from 36 to 39 months in 3 cohort studies [45–47], while one study had longer follow-up time (48 months) [44]. Therefore, this study was excluded from the sensitivity analysis. The pooled ORs were 7.15 (95%CI: 1.34, 38.01), suggesting that NLR had a greater effect on CVD risk in study with shorter follow-up time than study with longer follow-up time.

Egger's test (Coefficient = 6.96; P = 0.017) and funnel plot suggested publication bias; see Supplementary Figure 7B. A contour-enhanced funnel plot found that missing studies were in the nonsignificant area. Therefore, asymmetry in the funnel likely represents publication bias; see Supplementary Figure 7C.

## 4. Discussion

We conducted a systematic review and meta-analysis to assess the risk of NLR on CVD. Our study showed that high NLR was significantly associated with all CVD outcomes including CAD, ACS, stroke, and composite CVEs with pooled ORs ranging from 1.62 to 3.86. In addition, mean NLR was significantly higher in CVD patients than in controls, with USMDs ranging from 0.87 to 2.12.

For the effect of high NLR on CVD outcomes, the strongest association was found in composite CVEs with pooled OR of 3.86, while the highest mean difference of NLR was found in ACS outcome with USMD of 2.12. However, ACS was the most common outcome defined in composite CVEs. Therefore, this may imply that the NLR effect was strongest on ACS outcome although consistent associations were seen across all individual CVD events. This may be attributed to the fact that NLR was associated with both acute and chronic forms of the atherosclerosis process.

There are various possible mechanisms that can explain the relationship between elevated NLR and risk of cardiovascular events. Neutrophils secrete inflammatory mediators that can lead to vascular wall degeneration [50]. Conversely, lymphocytes regulate the inflammatory response and have an antiatherosclerotic role in which regulatory T-cell, a subclass of lymphocyte, may have an inhibitory effect on atherosclerosis [51]. Previous studies also showed that a low lymphocyte count served as an early marker of physiologic stress and systemic collapse secondary to myocardial ischemia mediated by cortisol release [52, 53]. Increased cortisol levels result in a reduction in the relative level of lymphocytes [54].

Prior evidence has shown that high NLR is significantly associated with progression of atherosclerosis [55] and is also an independent predictor of thin-cap fibroatheroma [56]. Neutrophil infiltration into atherosclerotic plaques has also been found in atherectomy specimens of ACS patients and may contribute to its destabilization [57]. Activated neutrophils are known to release a variety of proteolytic enzymes [58]; neutrophil elastase in particular has been shown to mediate both degradation of basement membrane constituents and endothelial damage [59].

The C-reactive protein (CRP), one of the inflammatory biomarkers, has been investigated and found to be strongly associated with the risk and prognosis of CVDs [48, 60–62]. It also has positive correlation with neutrophil, monocyte, and NLR [25, 60]. Previous studies illustrated that NLR could be a potential surrogate marker of systemic inflammation in its ability to predict hs-CRP [63] and CRP levels [64]. Therefore, NLR might not have only the direct effect on CVD risk but also have the indirect effect via CRP level. On the contrary, NLR might not be independently associated with CVD. The significant association between NLR and CVD may be confounded by CRP. However, to prove these hypotheses, further study that measured both NLR and CRP level is required.

Our study has some strengths. To the best of our knowledge, this is the first systematic review and meta-analysis to address the association between NLR and risk of CVD. The analysis was based on studies with relatively large sample sizes from diverse countries. All components of CVD including CAD, ACS, stroke, and TIA were included. In addition, the effect of high NLR and mean difference of NLR between CVD and non-CVD patients were estimated in our analysis.

However, some limitations could not be avoided. Most included studies were cross-sectional studies. Therefore, a causal link between NLR and risk of CVD could not be confirmed. Moreover, the eligible studies used various cut-off values for classifying high versus low NLRs. Although we recategorized these cut-off points based on aggregated data, there was still some overlap in cut-off points between low and high NLRs across studies. This discrepancy might cause moderate to high heterogeneity in some of our pooled estimates. Individual patient data meta-analysis would be more flexible for addressing this problem.

In addition, the Framingham Risk Score was shown to overestimate risk of coronary heart disease risk in a general population from several ethnicities [65]. However, a previous study demonstrated that NLR can independently predict CHD mortality and reclassify people in the intermediate risk category of the Framingham Risk Score to a higher category [66]. Currently, there are numerous models predicting incident CVD in the general population but many lack external validation [67]. Including NLR may help to improve the performance of CVD risk prediction model. Therefore, future research should explore on comparing existing CVD risk models with those including NLR.

## 5. Conclusion

The present systematic review and meta-analysis suggest that high NLR was associated with CAD, ACS, stroke, and composite cardiovascular events. Therefore, NLR should be considered when assessing the cardiovascular risk in the population.

## Figures and Tables

**Table 1:** Characteristics of included studies.

**Author**	**Year**	**Setting**	**Study Design**	**n**	**Population**	**Mean Age**	%**Male**	**Mean BMI**	%**DM**		%**HT**	%**DLP**	%**Smoke**	**Mean NLR**
*Coronary artery disease*														

Sonmez [10]	2013	Turkey	Cross-sectional	175	general	59.2	59.0	29.8	40.6		53.7	43.4	-	2.29
Naz [11]	2014	India	Case control	60	general	45	100	-	0		-	-	0	2.98
Mayyas [12]	2014	Jordan	Cross-sectional	128	general	57.4	57.0	29.6	44.5		77.3	62.5	35.2	2.67
Sari [13]	2015	Turkey	Cross-sectional	180	general	59.1	63.3	-	24.4		-	19.4	41.1	3.03
Aygün [14]	2015	Turkey	Cross-sectional	292	DM	56.3	88.4	-	100		64	55.5	33.2	2.00
Acar [15]	2015	Turkey	Cross-sectional	238	general	54.8	55.0	27.4	18.5		62.2	68.2	37.4	2.11
Verdoia [16]	2015	Italy	Cross-sectional	1,372	DM	69.0	69.2	28.3	100		80.9	60.3	22.9	-
Gungoren [17]	2015	Turkey	Case control	311	general	62.6	56.3	-	30.5		54.3	83.9	30.5	2.53
Yu [18]	2016	China	Cohort	942	general	64.9	58.7	24.4	36.2		68.3	13.2	37.6	2.92
Perl [19]	2016	Israel	Cross-sectional	522	general	66.0	73.0	-	36		72	80	43	3.21
Verdoia [20]	2016	Italy	Cross-sectional	3,728	general	67.6	69.3	-	36.8		71.2	55.4	26.6	-
Uysal [21]	2016	Turkey	Cross-sectional	194	general	62.5	69.0	-	26.3		29.4	12.4	38.7	2.59
Yilmaz [22]	2016	Turkey	Case control	80	general	59.5	52.5	-	14.4		22.5	-	17.5	2.51
Chittawar [23]	2017	India	Cross-sectional	265	DM	51.1	45.7	25.9	100		-	-	-	-
Guo [24]	2017	China	Cross-sectional	64	general	60.0	29.7	-	12.5		54.7	-	32.8	2.12
Sharma [25]	2017	India	Cross-sectional	324	general	-	-	-	-		-	-	-	1.31
Korkmaz [26]	2018	Turkey	Cross-sectional	113	general	57.0	78	-	-		-	-	-	2.41

*Acute coronary syndrome*														

Yu [18]	2016	China	Cohort	600	general	64.9	58.7	24.4	36.2		68.3	13.2	37.6	3.33
Zazula [27]	2008	Brazil	Cross-sectional	178	general	60.0	59.0	-	28		78	44	17	4.32
Nordestgaard [28]	2010	Denmark	Case cohort	699	general	68.4	63.4	27.0	10.1		32.8	14.3	28.9	-
Caimi [29]	2015	Italy	Case control	239	general	34.9	83.3	-	-		-	-	-	2.10
Qiu [30]	2016	China	Case control	72	DM	64.1	58.3	25.2	100		-	-	45.8	5.39
Nalbant [31]	2016	Turkey	Cross-sectional	284	general	70.3	63.7	-	-		-	-	-	5.42
Göktaş [32]	2018	Turkey	Cross-sectional	100	general	57.8	50.0	-	-		-	-	-	-

*Stroke*														

Saliba [33]	2015	Israel	Cohort	32,912	AF	73.2	48.4	-	32.8		74.7	59.2	-	-
Ertas [34]	2013	Turkey	Case control	126	AF	70.0	59.0	-	18.3		73	34.1	6.3	3.87
Celikbi-lek [35]	2014	Turkey	Case control	140	general	64.9	37.9	-	-		-	-	-	2.42
Akil [36]	2014	Turkey	Case control	85	general	52.3	58.8	25.0	-		5.9	-	23.5	2.38
Wang [37]	2015	China	Case control	100	general	57.4	53.0	-	-		-	-	-	1.74
Köklü [38]	2016	Turkey	Cross-sectional	254	general	69.5	70.5	26.0	42.5		76.8	68.5	31.5	2.62
Suh [39]	2017	South Korea	Cohort	24,708	general	51.8	49.9	-	17.9		22.1	26.9	18.3	-
Long [40]	2018	China	Cohort	210	Gastric cancer	67.1	72.9	-	-	-	-	-	-	4.24
Abete [41]	2018	Spain	Case control	102	general	60.1	64.7	30.6	18.6	57.8	39.2	54.9	-	2.15
Farah [42]	2018	Israel	Case control	230	general	68.4	59.0	-	-	-	-	-	-	3.24

*Composite outcomes*														

Tsai [43]	2007	Taiwan	Cross-sectional	1,872	DM	60.1	44.4	25.5	100	-	-	20.5	-	-
Azab [44]	2013	US	Cohort	338	DM	58.1	36.1	-	100	77.2	77.2	29	-	-
Solak [45]	2013	Turkey	Cohort	225	CKD	50.4	47.6	25.8	22.2	14.2	-	44	-	3.31
Abe [46]	2015	Japan	Cohort	86	ESRD	58.0	67.4	22.0	48.8	62.8	30.2	18.6	-	-
Quiros-Roldan [47]	2016	Italy	Cohort	3,454	HIV	38.1	71.3	-	7.4	7.9	34.7	65.6	-	1.80

AF, atrial fibrillation; BMI, body mass index; CKD, chronic kidney disease; DLP, dyslipidemia; DM, diabetes mellitus; ESRD, end stage renal disease; HIV, human immunodeficiency virus; HT, hypertension; NLR, neutrophil lymphocyte ratio.

**Table 2:** Pooled odds ratio of cardiovascular between high and low NLR.

Author	Year	NLR	CVD		Non-CVD		OR (95%CI)
		Cutoff	Low NLR	High NLR	Low NLR	High NLR	
*Coronary artery disease*							

Sari [13]	2015	2.30	-	-	-	-	1.51 (1.15, 2.00)
Aygün [14]	2015	2.05	40	56	109	87	1.75 (1.07, 2.88)
Acar [15]	2015	2.25	-	-	-	-	2.30 (1.19, 4.43)
Verdoia [16]	2015	2.03	349	783	103	137	1.69 (1.27, 2.24)
Yu [18]	2016	2.41	-	-	-	-	1.69 (1.48,1.94)
Verdoia [20]	2016	1.80	682	2172	251	633	1.26 (1.07, 1.49)
Chittawar [23]	2017	2.60	2	8	197	58	13.59 (2.81, 65.76)
Guo [24]	2017	2.45	-	-	-	-	2.01 (0.88, 4.63)
Sharma [25]	2017	2.13	-	-	-	-	1.49 (0.94, 2.37)

Pooled OR (95% CI)							1.62 (1.38, 1.91)

*Acute coronary syndrome*							

Yu [18]	2016	2.42	-	-	-	-	1.65 (1.43, 1.90)
Zazula [27]	2008	5.70	-	-	-	-	4.51 (1.51, 13.45)
Nordestgaard [28]	2010	-	-	-	-	-	1.52 (0.83, 2.79)
Caimi [29]	2015	2.19	39	43	11	12	1.01 (0.40, 2.55)
Göktaş [32]	2018	3.0	40	23	26	11	1.36 (0.57, 3.25)

Pooled OR (95% CI)							1.64 (1.30-2.05)

*Stroke*							

Saliba [33]	2015	3.15	649	332	24049	7882	1.56 (1.36, 1.79)
Ertas [34]	2013	3.17	20	19	64	23	2.64 (1.20, 5.81)
Akil [36]	2014	-	-	-	-	-	8.95 (1.88, 42.61)
Suh [39]	2017	3.00	23,530	219	936	23	2.64 (1.71, 4.08)

Pooled OR (95% CI)							2.36 (1.44, 3.89)

*Composite outcomes*							

Tsai [43]	2007	-	-	-	-	-	1.52 (0.96, 2.40)
Azab [44]	2013	2.40	20	206	23	89	2.66 (1.39, 5.09)
Solak [45]	2013	2.80	3	63	109	50	45.78 (13.71, 152.85)
Abe [46]	2015	3.67	10	26	33	17	5.05 (1.98, 12.86)
Quiros-Roldan [47]	2016	1.20	22	90	1091	2251	1.98 (1.24, 3.18)

Pooled OR (95% CI)							3.86 (1.73, 8.64)

CI, confidence interval; CVD, cardiovascular disease; NLR, neutrophil lymphocyte ratio; OR, odds ratio.

**Table 3:** Mean difference of neutrophil lymphocyte ratio between CVD and non-CVD patients.

Author	Year	CVD		Non-CVD		Mean differences (95% CI)
		N	mean [48]	N	mean [48]	
*Coronary artery disease*						

Sonmez [10]	2013	106	2.37 (0.89)	69	2.03 (1.56)	0.34 (-0.07, 0.75)
Naz [11]	2014	40	3.67 (1.62)	20	1.61 (0.84)	2.06 (1.44, 2.68)
Mayyas [12]	2014	60	2.61 (0.17)	68	2.72 (0.19)	-0.11 (-0.17, -0.05)
Sari [13]	2015	100	3.70 (2.60)	80	2.2 (1.7)	1.50 (0.87, 2.13)
Acar [15]	2015	71	2.50 (0.70)	90	1.90 (0.70)	0.60 (0.38, 0.82)
Gungoren [17]	2015	261	2.73 (1.07)	50	1.51 (0.42)	1.22 (1.05, 1.39)
Yu [18]	2016	691	3.62 (2.70)	251	2.14 (1.97)	1.48 (1.16, 1.79)
Perl [19]	2016	170	3.44 (2.90)	352	3.00 (2.50)	0.44 (-0.16, 1.04)
Uysal [21]	2016	152	2.77 (0.23)	42	1.97 (0.15)	0.80 (0.74, 0.86)
Yilmaz [22]	2016	40	2.51(0.65)	40	1.73 (0.71)	0.78 (0.48, 1.08)
Guo [24]	2017	31	2.93(1.82)	33	2.11 (0.79)	0.82 (0.13, 1.52)
Sharma [25]	2017	225	5.60(4.50)	99	4.30 (3.80)	1.30 (0.35, 2.25)
Korkmaz [26]	2018	63	2.66(0.86)	50	2.10 (0.53)	0.56 (0.30, 0.82)

USMD (95%CI)						0.87 (0.52, 1.22)

*Acute coronary syndrome*						

Yu [18]	2016	349	4.93 (3.15)	251	2.14 (1.97)	2.79 (2.38, 3.20)
Nordestgaard [28]	2008	133	4.77 (3.83)	45	3.00 (1.60)	1.77 (0.97, 2.57)
Caimi [29]	2015	123	2.38 (0.87)	116	1.82 (0.71)	0.56 (0.36, 0.76)
Qiu [30]	2016	38	8.10 (6.44)	34	2.37 (1.19)	5.73 (3.64, 7.82)
Nalbant [31]	2016	189	5.58 (6.60)	95	5.10 (7.60)	0.48 (-1.32, 2.28)

USMD (95%CI)						2.12 (0.70, 3.53)

* Stroke*						

Ertas [34]	2013	39	5.60 (3.40)	87	3.10 (2.10)	2.50 (1.35, 3.66)
Celikbilek [35]	2014	70	2.97 (0.53)	70	1.88 (0.40)	1.09 (0.96, 1.23)
Akil [36]	2014	38	3.10 (2.00)	47	1.80 (0.40)	1.30 (0.65, 1.95)
Wang [37]	2015	50	1.40 (0.83)	50	1.40 (0.41)	0.00 (-0.26, 0.26)
Köklü [38]	2016	115	3.09 (0.23)	139	2.23 (0.15)	0.86 (0.81, 0.91)
Long [40]	2018	70	5.51 (8.02)	140	3.60 (1.89)	1.91 (0.01, 3.82)
Abete [41]	2018	51	2.30 (0.50)	51	2.00 (0.30)	0.30 (0.14, 0.46)
Farah [42]	2018	200	3.44 (2.56)	30	1.89 (0.61)	1.55 (1.13, 1.97)

USMD (95% CI)						0.92 (0.60, 1.24)

CI, confidence interval; CVD, cardiovascular disease; USMD, unstandardized mean difference.

## Data Availability

The data used to support the findings of this study are included within the article.

## Supplementary Materials

Supplementary Materials Supplementary Appendix: search terms and search strategy used for Scopus and Medline database. Supplementary Table 1: risk of bias assessment (case-control study). Supplementary Table 2: risk of bias assessment (cohort and cross-sectional studies). Supplementary Figures: Supplementary Figure 1: high versus low neutrophil lymphocyte ratio and coronary artery disease. Supplementary Figure 2: mean differences of neutrophil lymphocyte ratio and coronary artery disease. Supplementary Figure 3: high versus low neutrophil lymphocyte ratio and acute coronary syndrome. Supplementary Figure 4: mean differences of neutrophil lymphocyte ratio and acute coronary syndrome. Supplementary Figure 5: high versus low neutrophil lymphocyte ratio and stroke. Supplementary Figure 6: mean differences of neutrophil lymphocyte ratio and stroke. Supplementary Figure 7: high versus low neutrophil lymphocyte ratio and composite cardiovascular events (CVEs).
