# Supplementary material for: Neutrophil Lymphocyte Ratio and Cardiovascular Disease Risk: A Systematic Review and Meta-Analysis
Source: Biomed Res Int. 2018 Nov 11;2018:2703518. doi: 10.1155/2018/2703518 (PMC6252240; doi:10.1155/2018/2703518)
Supplement: Supplementary Materials — Supplementary Appendix: search terms and search strategy used for Scopus and Medline database. Supplementary Table 1: risk of bias assessment (case-control study). Supplementary Table 2: risk of bias assessment (cohort and cross-sectional studies). Supplementary Figures: Supplementary Figure 1: high versus low neutrophil lymphocyte ratio and coronary artery disease. Supplementary Figure 2: mean differences of neutrophil lymphocyte ratio and coronary artery disease. Supplementary Figure 3: high versus low neutrophil lymphocyte ratio and acute coronary syndrome. Supplementary Figure 4: mean differences of neutrophil lymphocyte ratio and acute coronary syndrome. Supplementary Figure 5: high versus low neutrophil lymphocyte ratio and stroke. Supplementary Figure 6: mean differences of neutrophil lymphocyte ratio and stroke. Supplementary Figure 7: high versus low neutrophil lymphocyte ratio and composite cardiovascular events (CVEs). [file 2703518.f1.docx]

**SUPPLEMENTARY MATERIAL**

**Supplementary Appendix.** Search terms and search strategy used for Scopus and Medline database

**Supplementary Tables**

**Supplementary Table 1.** Risk of bias assessment (case-control study)

**Supplementary Table 2.** Risk of bias assessment (cohort and cross-sectional studies)

**Supplementary Figures**

**Supplementary Figure 1.**  High vs. Low neutrophil lymphocyte ratio and coronary artery disease (CAD)

**Supplementary Figure 2.** Mean differences of neutrophil lymphocyte ratio and coronary artery disease (CAD)

**Supplementary Figure 3.** High vs. Low neutrophil lymphocyte ratio and acute coronary syndrome (ACS)

**Supplementary Figure 4.** Mean differences of neutrophil lymphocyte ratio and acute coronary syndrome (ACS)

**Supplementary Figure 5.** High vs. Low neutrophil lymphocyte ratio and stroke

**Supplementary Figure 6.** Mean differences of neutrophil lymphocyte ratio and stroke

**Supplementary Figure 7.** High vs. low neutrophil lymphocyte ratio and composite cardiovascular events (CVEs)

**Supplementary Appendix.** Search terms and search strategy used for Scopus and Medline database

| **Scopus search** | | | **Medline search** | |
| --- | --- | --- | --- | --- |
| I | ALL(neutrophil) | 588,726 | Search neutrophil | 151030 |
|  | ALL(lymphocyte) | 1,475,845 | Search lymphocyte | 649395 |
|  | ALL( ratio ) | 4,290,028 | Search ratio | 972576 |
|  | ) (ALL ( neutrophil ) ) AND ( ALL ( lymphocyte ) ) AND ( ALL ( ratio ) ) | 22,739 | Search ((neutrophil) AND lymphocyte) AND ratio | 4844 |
|  | ALL("neutrophil lymphocyte ratio" | 5,979 | Search "neutrophil lymphocyte ratio" | 4844 |
|  | ((ALL ( neutrophil ) ) AND ( ALL ( lymphocyte ) ) AND ( ALL ( ratio ) ) ) OR  (ALL ( "neutrophil lymphocyte ratio" ) ) | 22,739 | Search ("neutrophil lymphocyte ratio") OR (((neutrophil) AND lymphocyte) AND ratio) | 4844 |
| O | ALL("cardiovascular disease" ) | 922,254 | Search "cardiovascular disease" | 121026 |
|  | ALL("coronary heart disease" ) | 402,301 | Search "coronary heart disease" | 47036 |
|  | ALL("coronary artery disease" ) | 504,253 | Search "coronary artery disease" | 110953 |
|  | ALL("myocardial infarction" ) | 724,732 | Search "myocardial infarction" | 226274 |
|  | ALL(angina) | 215,753 | Search angina | 67062 |
|  | ALL(stroke) | 1,033,055 | Search stroke | 294231 |
|  | ALL( cerebrovascular ) | 413,393 | Search cerebrovascular | 131001 |
|  | ( ALL ( "cardiovascular disease" )) OR (ALL( "coronary heart disease" )) OR (ALL( "coronary artery disease" )) OR (ALL( "myocardial infarction" )) OR (ALL(angina)) OR(ALL( stroke))OR(ALL(cerebrovascular )) | 2,555,772 | Search (((((("cardiovascular disease") OR "coronary heart disease") OR "coronary artery disease") OR "myocardial infarction") OR angina) OR stroke) OR cerebrovascular | 808471 |
| I  +  O | ( ( ALL ( "cardiovascular disease" ) ) OR ( ALL ( "coronary heart disease" ) ) OR ( ALL ( "coronary artery disease" ) ) OR ( ALL ( "myocardial infarction" ) ) OR ( ALL ( angina ) ) OR ( ALL ( stroke ) ) OR ( ALL ( cerebrovascular ) ) ) AND ( ( ( ALL ( neutrophil ) ) AND ( ALL ( lymphocyte ) ) AND ( ALL ( ratio ) ) ) OR ( ALL ( "neutrophil lymphocyte ratio" ) ) ) | 5,605 | Search ((("neutrophil lymphocyte ratio") OR (((neutrophil) AND lymphocyte) AND ratio))) AND ((((((("cardiovascular disease") OR "coronary heart disease") OR "coronary artery disease") OR "myocardial infarction") OR angina) OR stroke) OR cerebrovascular) | 461 |
|  | ( ( ALL ( "cardiovascular disease" ) ) OR ( ALL ( "coronary heart disease" ) ) OR ( ALL ( "coronary artery disease" ) ) OR ( ALL ( "myocardial infarction" ) ) OR ( ALL ( angina ) ) OR ( ALL ( stroke ) ) OR ( ALL ( cerebrovascular ) ) ) AND ( ( ( ALL ( neutrophil ) ) AND ( ALL ( lymphocyte ) ) AND ( ALL ( ratio ) ) ) OR ( ALL ( "neutrophil lymphocyte ratio" ) ) ) AND ( LIMIT-TO ( DOCTYPE , "ar" ) OR LIMIT-TO ( DOCTYPE , "re" ) ) AND ( LIMIT-TO ( SUBJAREA , "MEDI" ) ) AND ( LIMIT-TO ( LANGUAGE , "English" ) ) | 3,947 |  |  |
|  |  |  |  |  |

**Supplementary Table 1.** Risk of bias assessment (case-control study)

| Author | Year | Selection | | | | Comparability | Outcome | | | Total stars |
| --- | --- | --- | --- | --- | --- | --- | --- | --- | --- | --- |
|  |  | Is the case definition adequate? | Representativeness of cases | Selection of Controls | Definition of Controls | Comparability of cases and controls | Assessment of exposure | Same method of ascertainment for cases and controls | Non-response rate |  |
| Naz | 2014 | B(0) | A(1*) | A(1*) | A(1*) | A(0) B(1*) | A(1*) | A(1*) | B(0) | 6 |
| Gungo-ren | 2015 | B(0) | A(1*) | A(1*) | A(1*) | A(1*) B(1*) | A(1*) | A(1*) | B(0) | 7 |
| Caimi | 2015 | C(0) | A(1*) | A(1*) | A(1*) | A(0) B(0) | A(1*) | A(1*) | B(0) | 5 |
| Qiu | 2015 | B(0) | A(1*) | B(0) | A(1*) | A(1*) B(1*) | A(1*) | A(1*) | B(0) | 6 |
| Ertas | 2013 | B(0) | A(1*) | A(1*) | A(1*) | A(0) B(1*) | A(1*) | A(1*) | B(0) | 6 |
| Celikbilek | 2014 | C(0) | A(1*) | A(1*) | A(1*) | A(0) B(0) | A(1*) | A(1*) | B(0) | 5 |
| Akil | 2014 | C(0) | A(1*) | A(1*) | A(1*) | A(1*) B(1*) | A(1*) | A(1*) | B(0) | 7 |
| Wang | 2015 | C(0) | A(1*) | A(1*) | A(1*) | A(1*) B(0) | A(1*) | A(1*) | B(0) | 6 |
| Yilmaz | 2016 | A(1*) | A(1*) | B(0) | A(1*) | A(1*) B(1*) | A(1*) | A(1*) | B(0) | 7 |
| Abete | 2018 | B(0) | B(0) | B(0) | A(1*) | A(1*) B(0) | A(1*) | A(1*) | B(0) | 4 |
| Farah | 2018 | B(0) | A(1*) | A(1*) | A(1*) | A(0)B(0) | D(0) | A(1*) | B(0) | 4 |

**Supplementary Table 2.** Risk of bias assessment (cohort and cross-sectional studies)

| Author | Year | Selection | | | | Comparability | Outcome | | | Total stars |
| --- | --- | --- | --- | --- | --- | --- | --- | --- | --- | --- |
|  |  | Representativeness of cohort | Selection of the non-exposed cohort | Ascertainment of exposure | Outcome of interest | Comparability of cohorts | Assessment of outcome | Adequate duration of follow up | Adequacy of follow up |  |
| *Cohort study* |  |  |  |  |  |  |  |  |  |  |
| Nordestgaard | 2010 | A(1*) | A(1*) | A(1*) | A(1*) | A(1*) B(1*) | B(1*) | A(1*) | D(0) | 8 |
| Yu | 2016 | A(1*) | A(1*) | A(1*) | A(1*) | A(0) B(1*) | D(0) | A(1*) | A(1*) | 7 |
| Saliba | 2015 | A(1*) | A(1*) | A(1*) | A(1*) | A(0) B(0) | B(1*) | A(1*) | A(1*) | 7 |
| Azab | 2013 | B(1*) | A(1*) | A(1*) | A(1*) | A(1*) B(1*) | B(1*) | A(1*) | D(0) | 8 |
| Solak | 2013 | B(1*) | A(1*) | A(1*) | B(0) | A(1*) B(1*) | B(1*) | A(1*) | D(0) | 7 |
| Abe | 2015 | B(1*) | A(1*) | A(1*) | B(0) | A(1*) B(1*) | B(1*) | A(1*) | A(1*) | 8 |
| Quiros-Roldan | 2016 | B(1*) | A(1*) | A(1*) | A(1*) | A(0) B(0) | B(1*) | A(1*) | A(1*) | 7 |
| Suh | 2017 | B(1*) | A(1*) | A(1*) | A(1*) | A(1*) B(1*) | B(1*) | A(1*) | D(0) | 8 |
| Long | 2018 | C(0) | A(1*) | A(1*) | A(1*) | A(1*)B(0) | B(1*) | A(1*) | D(0) | 6 |
| *Cross-sectional* | | | | | | | | | | |
| Tsai | 2007 | B(1*) | A(1*) | A(1*) |  | A(1*) B(1*) | A(1*) | A(1*) |  | 7 |
| Zazula | 2008 | A(1*) | A(1*) | A(1*) |  | A(0) B(1*) | B(1*) | A(1*) |  | 6 |
| Sonmez | 2013 | A(1*) | A(1*) | C(0) |  | A(0) B(1*) | B(1*) | A(1*) |  | 5 |
| Mayyas | 2014 | B(1*) | C(0) | A(1*) |  | A(0) B(0) | B(1*) | A(1*) |  | 4 |
| Aygun | 2015 | B(1*) | A(1*) | A(1*) |  | A(1*) B(1*) | B(1*) | A(1*) |  | 7 |
| Acar | 2015 | B(1*) | A(1*) | A(1*) |  | A(0) B(1*) | B(1*) | A(1*) |  | 6 |
| Veroda | 2015 | B(1*) | C(0) | A(1*) |  | A(0) B(0) | B(1*) | A(1*) |  | 4 |
| Sari | 2015 | B(1*) | C(0) | A(1*) |  | A(0) B(1*) | B(1*) | A(1*) |  | 5 |
| Köklü | 2016 | A(1*) | A(1*) | A(1*) |  | A(1*) B(1*) | A(1*) | A(1*) |  | 7 |
| Uysal | 2016 | A(1*) | A(1*) | A(1*) |  | A(1*) B(1*) | A(1*) | A(1*) |  | 7 |
| Nalbant | 2016 | A(1*) | A(1*) | A(*) |  | A(1*) B(0) | B(1*) | A(1*) |  | 6 |
| Perl | 2016 | A(1*) | C(0) | A(1*) |  | A(0) B(0) | B(1*) | A(1*) |  | 4 |
| Veroda | 2016 | B(1*) | C(0) | A(1*) |  | A(0) B(1*) | B(1*) | A(1*) |  | 5 |
| Chittawar | 2017 | B(1*) | A(1*) | A(1*) |  | A(1*) B(1*) | B(1*) | A(1*) |  | 7 |
| Guo | 2017 | C(0) | A(1*) | A(1*) |  | A(1*) B(1*) | B(1*) | A(1*) |  | 6 |
| Sharma | 2017 | C(0) | A(1*) | A(1*) |  | A(1*) B(1*) | B(1*) | A(1*) |  | 6 |
| Göktaş | 2018 | C(0) | A(1*) | A(1*) |  | A(1*)B(1*) | B(1*) | A(1*) |  | 6 |
| Korkmaz | 2018 | C(0) | A(1*) | A(1*) |  | A(0) B(0) | A(1*) | B(0) |  | 3 |

**Supplementary Figure 1.**  High vs. Low neutrophil lymphocyte ratio (NLR) and coronary artery disease (CAD)

**Supplementary Figure 2.** Mean differences of NLR and CAD

**Supplementary Figure 3.** High vs. Low NLR and acute coronary syndrome (ACS)

**Supplementary Figure 4.** Mean differences of NLR and ACS

**Supplementary Figure 5.** High vs. Low NLR and stroke

**Supplementary Figure 6.** Mean differences of NLR and stroke

**Supplementary Figure 7.** High vs. low NLR and composite cardiovascular events (CVEs)
